# Multiple Osteomas in Middle Ear

**DOI:** 10.1155/2012/685932

**Published:** 2012-08-05

**Authors:** Yongxin Li, Qiuhuan Li, Shusheng Gong, Honggang Liu, Zilong Yu, Luo Zhang

**Affiliations:** ^1^Department of Otolaryngology, Head and Neck Surgery, Beijing Tongren Hospital, Capital Medical University, 1Dongjiao Minxiang, Beijing 100730, China; ^2^Department of Pathology, Beijing Tongren Hospital, Capital Medical University, 1Dongjiao Minxiang, Beijing 100730, China

## Abstract

Since the first description of middle ear osteomas by Thomas in 1964, only few reports were published within the English literatures (Greinwalid et al., 1998; Shimizu et al., 2003; Cho et al., 2005; and Jang et al., 2009), and only one case of the multiple osteomas in middle ear was described by Kim et al., 2006, which arose from the promontory, lateral semicircular canal, and epitympanum. Here we describe a patient with multiple middle ear osteomas arising from the promontory, incus, Eustachian tube, and bony semicanal of tensor tympani muscle. This patient also contracted the chronic otitis media in the ipsilateral ear. The osteomas were successfully removed by performing type III tympanoplasty in one stage.

## 1. Case Report

**Figure 1:**
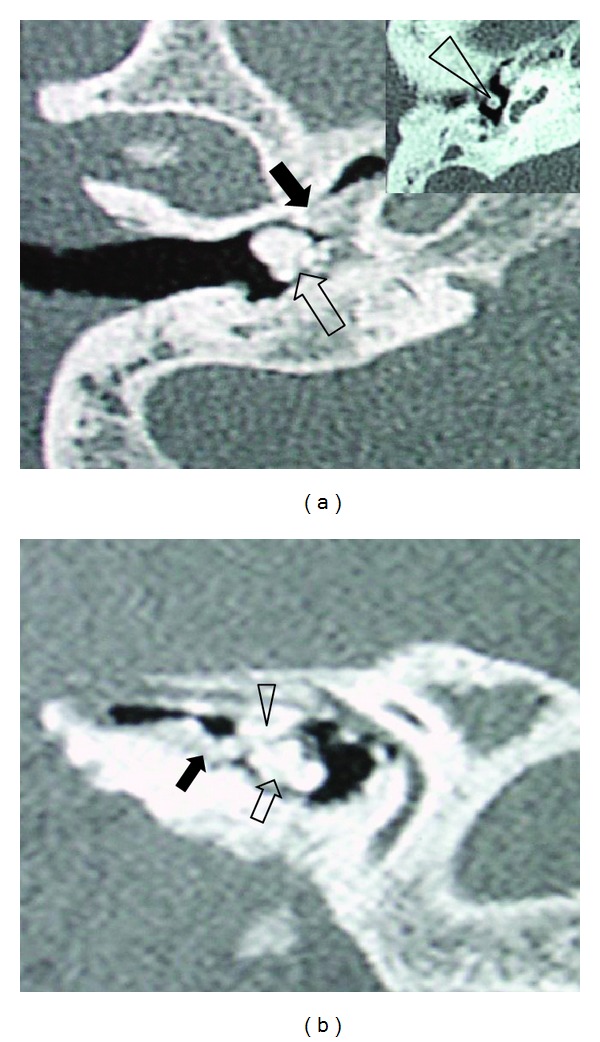
Axial (a) and parasagittal (b) computed tomography (CT) scan images of the patient. (a) One density pedunculated bony mass was on the promontory (hollow arrow), and another broad basal bony mass was on the Eustachian tube opening (black arrow), the third density bony mass located at the long crus of the incus (arrow head) (insert). (b) The density bony masses occupied the Eustachian tube opening (black arrow) and mesotympanum (hollow arrow) covering semicanal of tensor tympani muscle (arrow head).

A 52-year-old male patient presented with a progressive hearing loss and otorrhea over twenty years for the right ear. There was no history of ear trauma or otological surgery but with a history using Chlorine-ephedrines ear drops about seven years. Otoscopic examination showed white masses at the anteroinferior tympanic cavity via the perforated tympanic membrane. A pure tone audiogram showed a mixed hearing loss with an apparent air-bone conduct gap in the right ear. High-resolution-computed tomography (HRCT) scan of temporal bone demonstrated multiple high-density masses on the promontory, the Eustachian tube, the bony semicanal of tensor tympani muscle, and the long crus of incus (Figure 1). These masses obstructed the Eustachian tube opening. There was no evidence of erosion of the promontory.

**Figure 2:**
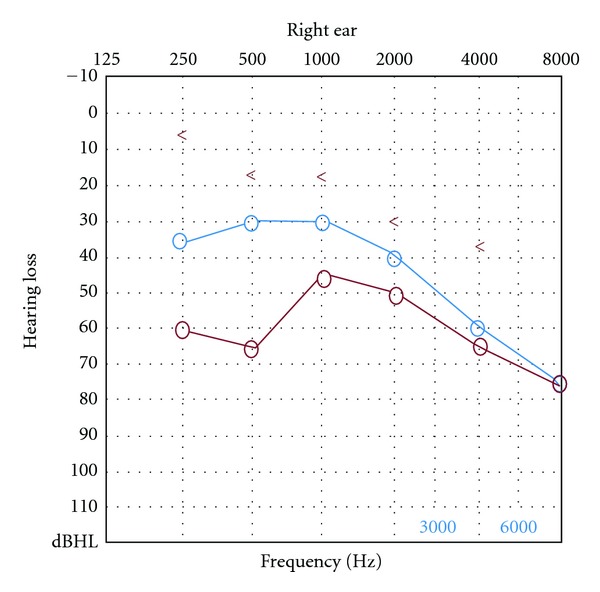
Pure tone audiogram of preoperative and postoperative hearing for the operative ear (red line: preoperative air conduction threshold; blue line: postoperative air conduction threshold).

**Figure 3:**
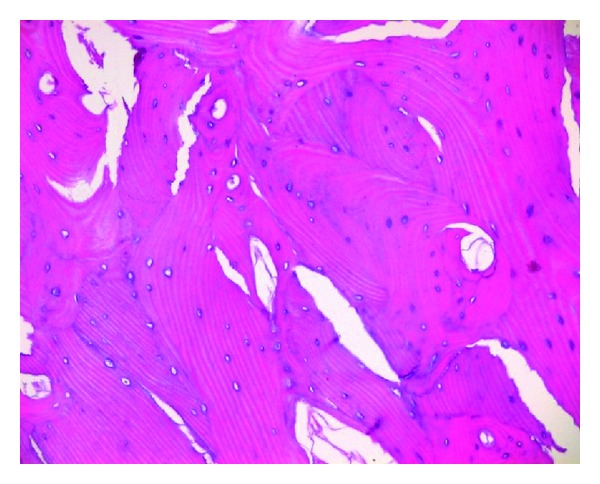
Hematoxylin-eosin staining of the osteoma. Whirlpool-like array of the lamellar bone which contains abundant fibrovascular channels and osteocytes (×10).

The patient underwent canal wall up mastoidectomy and tympanoplasty under general anesthesia. A series osteomas were removed during the surgical procedures. Firstly, a very small bony mass located at the long crus of the incus (about diameter 1.5 mm) was found and the incus was elevated. Then a huge rock-hard white mass with pedunculate was detached from the promontary (medial wall of hypotympanum), en bloc. At last, several egg-stone-like bony masses covered the bony semicanal of tensor tympani muscle and the Eustachian tube were detached and/or drilled away, respectively. After removal of these osteomas, a type III tympanoplasty was performed (the prosthesis from sculptured mastoid cortical bone). Average 20 dB hearing improvement at 500, 1000, 2000 Hz was obtained six months after surgery (Figure 2). Histopathologic examination of the mass showed a tumor of lamellar bone (Figure 3).

## 2. Discussion

While most of the temporal bone osteomas arise from the external auditory canal [1], osteomas in the middle ear are extremely rare. To our knowledge, only one multiple middle ear osteomas has been reported in the English literature [2]. In the current report, the osteomas were firmly attached to the promontory, the Eustachian tube, the semicanal of tensor tympani muscle, and the long crus of incus.

The most common symptom of osteomas in the middle ear is a progressive conductive hearing loss, occasionally accompanying otorrhea, and/or tinnitus. The mechanism responsible for this hearing loss involves ossicular chain fixation [3], dislocation [4], round window obliteration by the osteoma [5], or impingement on the tympanic membrane [6]. The obstruction of eustachian tube by osteomas may cause chronic otitis media or otorrhea, and recurrent otitis media with effusion might cause the elevation of the bone conduction threshold. In most cases, the diagnoses were confirmed by CT scan or visual inspection via the perforated tympanic membrane or confirmed in surgical exploration. Although some cases of middle ear osteoma may be asymptomatic [7], and there was no progression in tumor size after 9 years of followup in the middle ear osteomas [8], surgical intervention is still recommended for symptomatic lesions, such as osteomas with ossicular chain involvement, eustachian tube obstruction, otic capsule erosion, round window obstruction, or accompanying otorrhea.

The etiology of osteomas in the middle ear remains unclear. There is some evidence of congenital origin [9], autosomal dominant disease [10], familial osteoma [11], or inflammatory origin [3] in some osteomas. In our case, the patient has a chronic otitis media and long-term usage of ephedrine as ear drops, which may be a possible etiology of osteomas.
